# Patient experience of medication administration and development of a Patient Experience and Preference Questionnaire (PEPQ) for patients with advanced or metastatic cancer

**DOI:** 10.3389/fphar.2024.1310546

**Published:** 2024-03-27

**Authors:** Anne Skalicky, Bryan Bennett, Judith Raimbourg, Sara Lonardi, Julia Correll, Iwona Lugowska, Matthew Dixon, Nashmel Sargalo, Mona L. Martin

**Affiliations:** ^1^ Evidera, Seattle, WA, United States; ^2^ Bristol Myers Squibb, Uxbridge, United Kingdom; ^3^ Department of Medical Oncology, Institut de Cancérologie de l’Ouest, St Herblain, France; ^4^ Department of Oncology, Veneto Institute of Oncology IOV-IRCCS, Padova, Italy; ^5^ Maria Sklodowska Curie National Cancer Institute of Oncology, Warsaw, Poland; ^6^ Bristol Myers Squibb, Lawrenceville, NJ, United States; ^7^ Evidera, London, United Kingdom

**Keywords:** subcutaneous injection, intravenous infusion, patient experience, patient satisfaction, Patient Experience and Preference Questionnaire, oncology, qualitative interviews

## Abstract

**Introduction:** A better understanding of patient experience of intravenous (IV) or subcutaneous (SC) routes of administration is fundamental to providing optimal administration of medical therapies to oncology patients. The objective of this study was to examine patient experiences of IV and SC treatment with nivolumab and confirm the relevance of item concepts in the Patient Experience and Preference Questionnaire (PEPQ). The PEPQ is a clinical outcomes’ assessment instrument developed to obtain patient-centric data and understand the experience with IV and SC treatment administration.

**Methods:** Embedded qualitative interviews were conducted with a subset of participants from three treatment cohorts with metastatic non-small-cell lung cancer (NSCLC), renal cell carcinoma (RCC), unresectable or advanced metastatic melanoma, hepatocellular carcinoma (HCC), or colorectal cancer (CRC) from the CA209-8KX clinical trial. Concept elicitation interviews were conducted within 14 days of the initial treatment cycle and patient experiences with IV and SC treatment administration were assessed. Concepts from interviews were mapped to the PEPQ version 1.0 questions to assess relevance and convergence of concepts.

**Results**: Interviews were conducted with 43 trial participants from clinical sites opting to participate from six countries (Argentina, France, the Netherlands, Poland, Spain, and New Zealand). The mean age of sub-study participants was 66 ± 11.3 years (range 24–80 years), and 67.4% (*N* = 29) were male. Sub-study participants with experience of SC most frequently reported symptoms or signs of injection-related redness (27.9%), itching (14.0%), and pain (of needle), and described the pain as pricking, stinging, or tingling (11.0% each). The amount of pain and time burden were widely endorsed as important factors for satisfaction and related to the route of medication administration. For 11 sub-study participants with experience with both IV and SC treatments, 10 (90.9%) preferred SC over IV treatment administration.

**Conclusion:** This study summarizes the experience and satisfaction of receiving IV or SC treatment and confirms the relevance of the PEPQ in a subgroup of CA209-8KX clinical trial participants with metastatic NSCLC, RCC, melanoma, HCC, and CRC. Participant treatment experience and satisfaction with the route of medication mapped to the PEPQ question content support the relevance of PEPQ v2.0 in clinical trials as a self-report measure.

## 1 Introduction

Nivolumab is a programmed death-1 inhibitor that has shown demonstrable clinical benefits in various types of tumors. It is globally approved for intravenous (IV) administration, both as a monotherapy and in combination with other immuno-oncology therapies. These therapies have revolutionized the cancer treatment landscape (Bristol-Myers Squibb Company; Lonardi et al., 2021; Anderson et al., 2019). Subcutaneous (SC) delivery decreases the burden associated with IV administration for patients, healthcare providers, and healthcare systems related to time and resources. SC administration can reduce the time spent waiting for and receiving treatment as it shortens injection times, removes the need for IV infusion ports, decreases time spent on infusions, reduces provider and facility time to deliver cancer care, and offers cost-saving advantage compared to IV (Anderson et al., 2019).

A better understanding of patient preference for the route of administration of immuno-oncology therapies by IV or SC is fundamental in providing optimal medical therapies and may result in better outcomes for patients with cancer (Shingler et al., 2014). Measuring patient-reported experience of the route of treatment administration in controlled studies has been more common in chronic conditions (Overton et al., 2021) and, with many commonly including *de novo* questionnaires, or single item numeric or visual rating scales (Stoner et al., 2014; Callis Duffin et al., 2016; Schiff et al., 2016; Stauffer et al., 2018), the preference for the route of administration of oncology therapies is not readily comparable to routes of administration of therapies for patients with chronic disorders, given oncology patients often are on treatment for a limited period of time and are administered treatments typically during hospital visits, by a health professional.

To better understand experiences with and preferences for routes of administration, existing patient-reported outcome (PRO) questionnaires assessing treatment satisfaction were examined. No suitable PRO questionnaire that included patient experience and preference for routes of medication administration was identified or publicly available at the time of the CA209-8KX trial. Existing questionnaires like the European Organization for Research and Treatment of Cancer in patient satisfaction questionnaire (EORTC QLQ-SAT32) were considered to be more focused on general aspects of patient treatment satisfaction rather than satisfaction with the route of treatment administration (Brédart et al., 2010). Thus, the *de novo* Patient Experience and Preference Questionnaire (PEPQ) was developed by a team of PRO and clinical experts through a combination of a targeted literature review and clinical considerations about potentially relevant item concepts for inclusion for measuring satisfaction of the route of treatment administration (Abetz et al., 2005; Stoner et al., 2014). The PEPQ includes questions on patient experience of the treatment, and acceptability and satisfaction with the route of treatment administration.

### 1.1 Background on the clinical trial

A phase I/II multi-tumor clinical trial of an SC formulation of nivolumab monotherapy (CA209-8KX) was conducted to evaluate its safety and tolerability with and without recombinant human hyaluronidase PH20 (rHuPH20) (NCT03656718) (Lonardi et al., 2021; Lonardi et al., 2022; ClinicalTrials, 2023). Results from this trial have been used to establish the SC dose of nivolumab for current and potential future studies. The target population for this clinical trial included patients with advanced or metastatic tumors approved for the treatment with nivolumab IV monotherapy: non-small-cell lung cancer (NSCLC), renal cell carcinoma (RCC), unresectable or metastatic melanoma, hepatocellular carcinoma (HCC), and microsatellite instability-high or mismatch repair-deficient colorectal cancer (CRC).

An exploratory objective of the trial was to administer the PEPQ and obtain patient-centric data to understand patient experience and IV and SC administration of nivolumab to capture concepts relevant to the experience and satisfaction with the route of medication administration.

To obtain patient feedback, an embedded qualitative interview sub-study was conducted with a subset of CA209-8KX clinical trial participants to examine the experience with IV/SC treatment to support a deeper understanding of patients’ experience of the route of study medication administration. The study did not aim to compare or contrast SC or IV, but included both participants as was possible to make sure the concepts of satisfaction with treatment and route of medication administration could be captured. An exploratory objective of the qualitative interview study was to confirm concepts captured in the PEPQ. Embedded interviews during clinical trials are a recommended method for obtaining more detail on patients’ perspective on treatment benefits and clinical outcome assessment development within the context of a clinical study (Food and Drug Administration, 2022a).

## 2 Methods

This sub-study received local institutional review board (IRB) and ethics committee (EC) approval through CA209-8KX clinical trial sites and was performed in accordance with good clinical practice and applicable regulatory requirements.

### 2.1 Study population

All CA209-8KX trial participants enrolled in the randomized controlled trial were 18 years or older and had histologic or cytologic confirmation of advanced (metastatic and/or unresectable) solid tumors. CA209-8KX clinical trial participants with NSCLC, RCC, melanoma, HCC, or CRC from three treatment cohorts (parts C, D, and E) were eligible for the optional interview sub-study. Participants in cohort parts A and B crossed over from nivolumab IV dosing (4 weeks after last IV dose) to part C; nivolumab 1200 mg was administered SC with rHuPH20 (manually using a syringe every 4 weeks). Participants in parts D (1200 mg SC nivolumab with rHuPH20 every 4 weeks) and E (600 mg SC nivolumab with rHuPH20 every 2 weeks) only had experience with SC treatment (Ascierto et al., 2022; Jackson et al., 2022; Lonardi et al., 2022).

#### 2.1.1 Study measures and procedures

CA209-8KX clinical sites participating in parts C, D, and E were approached to recruit participants for the sub-study. Clinical sites opting to participate in the sub-study approached participants in CA209-8KX parts C, D, and E during the trial consenting process and invited them to participate in the optional one-time telephone interview. To be eligible for the sub-study, participants had to be interviewed on telephone within 14 days of the initial parts C, D, or E treatment cycle; agree to have the interview audio-recorded; and provide informed consent. Each trial participant was given oral and written information about the nature, purpose, possible risk, and benefit of the interview sub-study and provided with the informed consent form (ICF) to review, with the opportunity to ask any questions of the local site coordinators. As the interview window approached, the interviewer contacted the site staff to confirm consent and contacted the trial participant to schedule the interview. Clinical staff administered a paper version of the PEPQ along with other trial-related assessments.

#### 2.1.2 Patient experience and preference questionnaire

**FIGURE 1:**
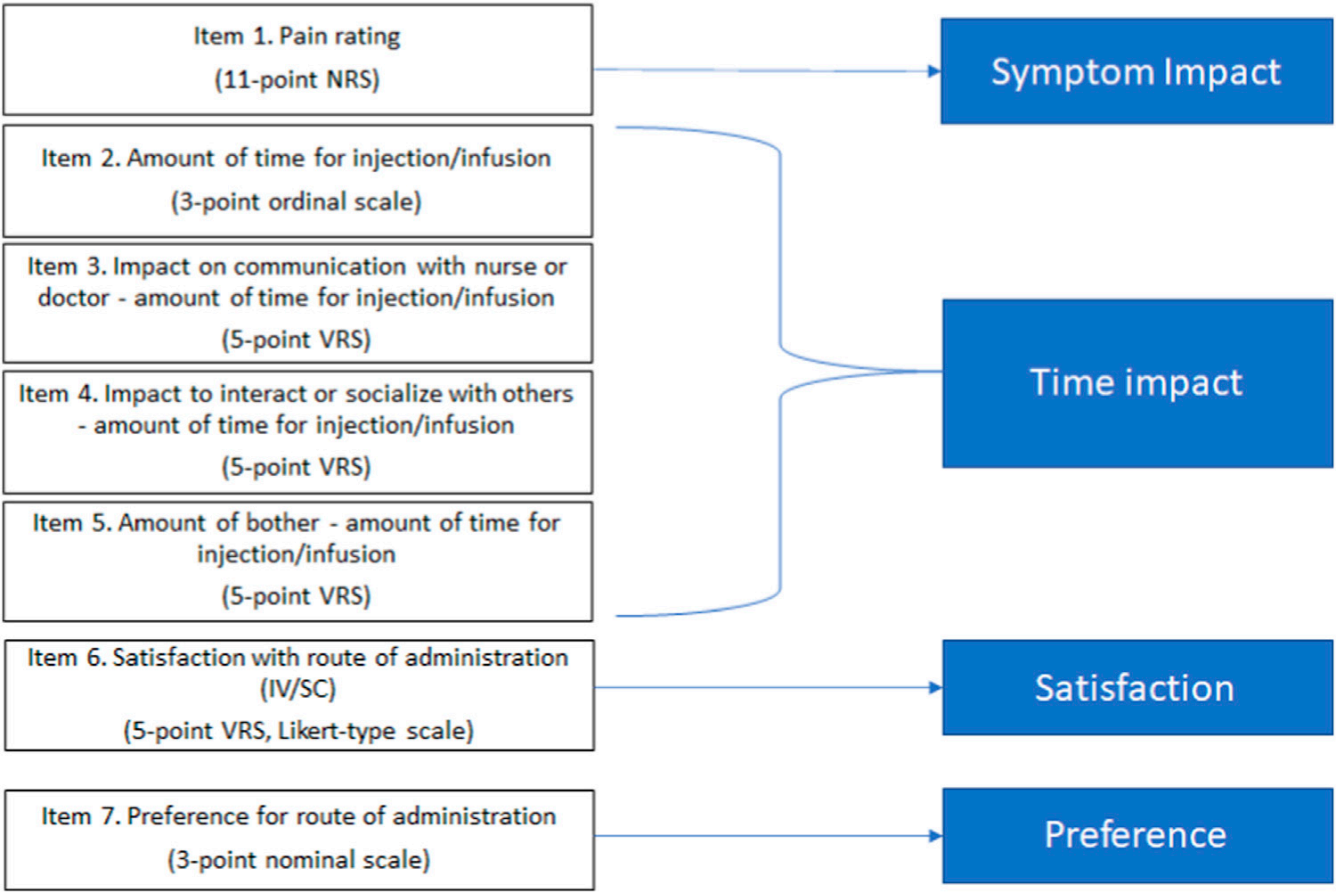
Conceptual framework PEPQ version 2.0.

The PEPQ version 1.0 (v1.0) is an eight-question interview-administered questionnaire capturing the current assessment of patient experience and preferences regarding the acceptability of the route of administration, treatment-related symptoms, and satisfaction with the treatment. The PEPQ v1.0 included questions to assess experience during injection or infusion, including a pain rating, a symptom checklist, time to administer study medication, impact of the study medication administration on patient’s time, impact of the study medication administration on patient’s time to speak to a nurse or doctor and socialize, level of patient disturbance with the time to administer the study medication, overall satisfaction with the treatment, and preference for IV or SC treatment (Figure 1). The PEPQ v1.0 was envisioned as interviewer-administered primarily due to the symptom checklist. The PEPQ v1.0 was available in native languages specific to clinical trial sites. Each item is scored and interpreted individually, with no summation for the total score. The questionnaire was administered by site staff immediately following completion of the injection or infusion on day 1 of treatment cycles 1 (IV) and 2 (SC) in parts A and B, and on day 1 of cycle 1 (SC) for participants in parts C, D, and E.

#### 2.1.3 Qualitative interview guide

Qualitative interviews were performed with participants to gather insights beyond those captured in the PEPQ. The interview guide followed a semi-structured method of inquiry that combined a set of open-ended questions to elicit spontaneous descriptions of the participants’ symptoms and treatment-related experiences and several close-ended questions to obtain ratings of symptom severity and bother. The interviewer probed further for additional details if not mentioned by participants and to gather further details about experiences with the infusion or injection event and assess whether there were any missing concepts in assessing satisfaction with the route of medication administration. The interview guide was translated into French, Dutch, Polish, and Spanish.

#### 2.1.4 Interview methods

Senior investigators (MM and NS) trained the interview team on the objectives, procedures, and content of the interview guide. Experienced and native language-speaking qualitative interviewers conducted the interviews. All interviews were conducted *via* telephone. Each interview was audio recorded with permission obtained through the informed consent process that was conducted at the site, and permission to record the interview was confirmed at the onset of the interview session. Participants did not receive remuneration for their participation in the sub-study.

Participants in cohort part C (coming from earlier phases of the trial and having recent experiences with IV treatment) were asked to talk about their experiences with and preferences of IV and SC routes of study medication administration. Parts D and E cohort participants were not asked the preference question because they only had experience with SC administration during their treatment phase.

Non-English interview audio files were simultaneously interpreted by professional linguists creating an English audio file for transcription. English audio files were transcribed. All transcripts were reviewed for personally identifying information, which was redacted, and the English transcripts were reviewed against the English audio files to ensure accurate transcription.

#### 2.1.5 Analysis

The analysis for this study used a mixed-methods approach. Quantitative data were derived from interviewer-administered questions during the interview process and documented onto a data capture form. These included ratings of severity- and symptom-related bother and other variables, including timing of symptoms and whether interview sub-study participants spoke about symptoms or impacts spontaneously.

A coding framework was developed based on the interview guide and study objectives. During the coding of transcripts, new codes were added to the coding framework as needed to capture emerging information. Eight coders were trained by a qualitative data manager (JC) and instructed on using the coding framework. Qualitative data from participants’ open-ended responses were coded from the interview transcripts. Coders identified concepts mentioned by participants during the interview and assigned codes based on the coding framework. ATLAS.ti v9 software was used to support the coding process and organize the codes based on similarity of content (Friese, 2019). To demonstrate consistency of the coded data, the inter-coder agreement was evaluated in a random sample of 10% of the final interview transcripts. These selected transcripts were independently dual-coded (each selected transcript coded by two separate coders). Each dual-coded pair of coders was compared for percent agreement in code assignment. The agreement was determined as the percentage of concepts that were given the same code across two coders with a minimum threshold of 90% agreement (Patrick et al., 2011a).

Coded concepts were exported from ATLAS.ti to allow for code counts specific to the PEPQ and related quotations. Coded data were organized to present participant feedback on PEPQ concepts. Saturation of concept was evaluated by grouping transcripts chronologically and breaking into smaller transcript groups for the comparison of elicitation of symptom and impact concepts. Each subsequent transcript group was compared to the prior group to identify the appearance of new codes (representing new information). If new concepts appeared in the last transcript group, saturation was considered incomplete for those emergent concepts (Rothman et al., 2009; Patrick et al., 2011a; Patrick et al., 2011b).

Relevance of the PEPQ item concepts was evaluated by comparing the concepts elicited during the interview with the question concepts in the PEPQ. The interview results from participants in cohort parts C, D, and E were analyzed together, focusing on the relevance of the patient experience with the route of treatment administration and the content of the PEPQ. The denominator for participant feedback is noted throughout and may not sum to the total sample size due to the semi-structured nature of the interview.

## 3 Results

### 3.1 Participant characteristics

The sub-study sample composed of 43 completed interviews: 11 part C participants, 15 part D participants, and 17 part E participants. Tumor types of interview participants were 33% RCC (*n* = 13), 23% CRC (*n* = 10), 19% NSCLC (*n* = 8), 16% HCC (*n* = 7), and 16% MM (*n* = 5). The mean age of participants was 66 years old ±11.3 standard deviation (range 24–80 years), and 67.4% (N = 29) were male. Participants reported time since diagnosis (with various tumor types) ranged between a half year to 18 years, with an average of 5 years. All but one participant (97.7%) reported receiving SC treatment in the belly. Out of the eligible CA209-8KX clinical sites from 11 countries, sub-study interviews were conducted in the following six countries: Argentina, France, the Netherlands, Poland, Spain, and New Zealand. Not all clinical sites chose to participate in the opt-in qualitative interview sub-study due to staffing, low patient numbers, or other reasons. New Zealand had the largest number of interview participants (*n* = 23), followed by seven participants from Poland, six participants from France, three participants from the Netherlands, and two participants from Argentina and Spain each. Participant characteristics are provided in Table 1.

**TABLE 1:** Participant characteristics.

Characteristic	Total (N = 43)
Age (years)	
Mean (SD)	65.6 (11.3)
Median (range)	68 [24 to 80]
Gender, N (%)	
Male	29 (67.4)
Female	14 (32.6)
Country, N (%)	
Argentina	2 (4.7)
France	6 (14.0)
Netherlands	3 (7.0)
New Zealand	23 (53.5)
Poland	7 (16.3)
Spain	2 (4.7)
Years since diagnosis	
Mean (SD)	5.1 (5.5)
Median (range)	2.8 [0.5 to 18]
Trial participation, N (%)	
Part C (960 mg SC nivolumab without rHuPH20)	11 (25.6)
Part D (1200 mg SC nivolumab with rHuPH20)	15 (34.9)
Part E (600 mg SC nivolumab with rHuPH20, every 2 weeks)	17 (39.5)
Tumor type, N (%)	
Non-small-cell lung cancer	8 (19)
Renal cell carcinoma	13 (30)
Unresectable or metastatic melanoma	5 (12)
Hepatocellular carcinoma	7 (16)
Colorectal cancer	10 (23)
SC injection location, N (%)	
Belly	42 (97.7)
Thigh	1 (2.3)

Abbreviations: rHuPH20, recombinant human hyaluronidase; SC, subcutaneous; SD, standard deviation.

On average, interviews were 30 min in length. Inter-coder agreement was at least 90%, demonstrating consistency in the coded data. Saturation was assessed using symptom and impacts reported by participants. Saturation findings identified that approximately 40% of the symptom and impact concepts mentioned by participants were elicited in the first transcript group (interviews 1–6, part C), followed by 11% in the second (interviews 7–12, parts C and D), and 16% in the third transcript group (interviews 13–18, parts C and D). Ten percent of concepts arose in the fourth group (interviews 19–24, part D), 19% in the fifth transcript group (interviews 25–30, parts D and E), and 3% in the sixth transcript group (interviews 31–36, part E). Only two new concepts appeared in the seventh and final transcript group (interviews 37–43, part E): pinching pain (additional pain description) and bone pain (additional pain location). Pain as a larger conceptual sub-domain had previously arisen in the first and second transcript groups.

The most frequently reported symptoms or signs occurring immediately after infusion or injection were injection-related redness (*n* = 12/43, 27.9%), itching (*n* = 6/43, 14.0%), pricking pain (of needle), and stinging or tingling (*n* = 5/43, 11.0% each). Participants considered their pain experience during or after the injection broadly as pain from either the needle or from drug administration. Pain/discomfort symptoms and tiredness after infusion or injection were rated (on a 0–10 numeric rating scale) as the most severe symptoms (mean values 5.5 and 5.4, respectively).

### 3.2 Concept elicitation to confirm experience and satisfaction with IV or SC administration

During interviews, participants were asked about the overall experience of the IV or SC medication administration, specifically probing on symptoms experienced, the time burden of the IV infusion or SC injection, and their level of satisfaction of the injection or infusion. The focus of interviews was to assess the concepts in the PEPQ of in relation to the specific route of treatment medication administration. Participant feedback on treatment experiences were mapped to the PEPQ question content to demonstrate concept relevance as outlined in Table 2 and described further below.

**TABLE 2:** Participant feedback confirming concepts of the Patient Experience Preference Questionnaire.

Patient Experience and Preference Questionnaire question	Concepts reported during interview	Sub-study interview sample, N (%) out of 43	Sample quotes from sub-study interview transcripts
**Question 1:** Using a scale from 0 to 10, where 0 represents “no pain or discomfort at all” and 10 represents “pain or discomfort that is as bad as you can imagine,” please rate the overall amount of pain or discomfort that you experienced during the injection or infusion of the study medication today	Pain, pricking pain, ache or dull sensation, discomfort, burning, stinging, and tenderness	N = 17 (39.5)	*I had slight discomfort. A wee bit of discomfort in my tummy area [around injection]. …I felt a little bit of pressure, a little bit of pain—not intense pain—around the injection point.* (0014–00015_SC-IV_Part C_RCC) *I felt rather intense pinching at the site where the drug was injected, and then it all passed. It was a bit intense, but short lasting.* (0019–00188_SC_Part E)
**Question 2:** Please tell me any words that you would use to describe any feelings or sensations that you may have experienced at any point during the injection or infusion of your study medication today. • Pain • Warm • Cold • Burning • Stinging • Itching • Tingling • Swelling • Lump • Tenderness • Did not report any sensations	Pain	N = 2 (4.7)	*Driv[ing] home*, *I felt a little bit of pressure, a little bit of pain—not intense pain—around the injection point.* (0014–00015_IV-SC_Part C_RCC)
	Pricking pain (of needle)	N = 5 (11.6)	*It was a similar sort of thing to the needle going in, just sort of a brief—almost a stab—a bit of a pinprick.* (0018–00028_IV-SC_Part C_NSCLC)
	Pinching pain	N = 1 (2.3)	*I felt rather intense pinching at the site where the drug was injected, and then it all passed. It was a bit intense, but short lasting.* (0019–00188_SC_Part E)
	Ache or dull sensation	N = 2 (4.7)	*I could feel it going in, but it was not necessarily painful. It was like a dull feeling.* (0014–000136_SC_Part D_RCC)
	Discomfort	N = 1 (2.3)	*Slight discomfort. A wee bit of discomfort in my tummy area [around injection].* (0014–00015_ IV-SC _Part C_RCC)
	Warm or hot sensation	N = 3 (7.0)	*Definite difference in the temperature of my skin from one side of my abdomen to the other. Warmth.* (0018–00028_IV-SC_Part C_NSCLC)
	Cold sensation	N = 2 (4.7)	*When the liquid went in, it was a wee bit cold.* (0014–00170_SC_Part E)
	Burning	N = 3 (7.0)	*It would burn a little slight bit.* (0014–00021_IV-SC_Part C_CRC)
	Stinging	N = 5 (11.6)	*Well, there was a tiny, tiny little bit of sting. It was probably at least from the needle going in, when I say it started, the infusion.* (0018–00028_IV-SC_Part C_NSCLC)
	Irritation	N = 1 (2.3)	*Irritation [at injection site]… it was mainly sort of more an irritation really.* (0014–00021_IV-SC_Part C_CRC)
	Itching	N = 6 (14.0)	*After the second injection, I had—it was an itch. So, I scratched the itch, and it stopped.* (0019–00168_SC_Part E_UC)
	Tingling	N = 5 (11.6)	*All around the injection area, sort of tingling…. and then it just faded away, yes.* (0014–00021_IV-SC_Part C_CRC)
	Swelling	N = 1 (2.3)	*[Swelling felt like] a little bit of tightness on my skin. One side of my stomach was… swollen compared with the other.* (0018–00028_IV-SC_Part C_NSCLC)
	Lump	N = 3 (7.0)	*You could sort of feel a little puddle [under skin], but there was no pain, no sensation, no burning, no nothing.* (0015–00007_NSCLC) *You know, now it gets hard—a little bit hard around the… where the injection is, yeah. But tha't’s all gone.* (0015–00121_NSCLC)
	Thickening	N = 1 (2.3)	*What remains is like a very small thickening, like the thickness of a sheet of paper.* (0019–00168_SC_Part E_UC)
	Tenderness	N = 3 (7.0)	*[Tenderness] it’s very slight.* (0014–00021_IV-SC_Part C_CRC)
	Sensitivity	N = 1 (2.3)	*Just a bit sensitive, that you know that you’ve had an injection there. Just like when you have got an insect bite.* (0039–00167_IV-SC_Part C_HCC)
	Did not report any sensations	N = 15 (34.9)	*No, no, no pain whatsoever.* (0039–00084_IV-SC_Part C_HCC)
*Question 3: Which statement best describes the amount of time it took for your study medication to be injected/infused? a) Injection/infusion of the study medication took less time than I expected. b) Injection/infusion of the study medication took an acceptable amount of time. c) Injection/infusion of the study medication took longer than I expected.	Longer than expected	N = 6 (13.9)	*No, I thought it was going to be faster. I thought it was going to be less than 1 minute. It was a large volume of medicine, but the nurse did great because she was injecting very slowly, but I did not feel anything.* (0016–00103_SC_Part D_RCC) *Well, it took longer. The lady who did it, did it very slowly, and she kept asking every once in a while if I feel any pain or anything and I said, no, no.* (0019–00178_SC_Part E_RCC)
	About the same as expected (or not specified)	N = 17 (39.5)	*It was what I expected. I have been kept very well-informed by my trial nurse, and she had explained that it would take between 4 and 6 min.* (0018–00028_IV-SC_Part C_NSCLC) *Well, I had been surprised to learn that it was only going to take about 5 min, so I was really pleased to find that that was correct.* (0014–00113_SC_Part D_CRC)
	Less time than expected	N = 19 (44.2)	*No, no, I was quite surprised that it took so little time to what I had expected. I thought it would take 10 min to a quarter of an hour at least.* (0014–00024_IV-SC_Part C_RCC) *Well, they did tell me in advance that it would take 5 min. It surprised me that having had previous things through IV and that, that [SC] was such a short space of time.* (0014–00136_SC_Part D_RCC)
**Question 4:** Did the length of time to administer the study medication impact the amount of time you had to speak to your nurse or doctor about your illness or other concerns? a) Not at all b) A little bit c) Somewhat d) Quite a bit e) Very much f) Not applicable	No impact	N = 43 (100.0)	*[Tx did not affect time with providers]. They may go away and attend someone else, but then they will come back again. That goes on for about a good half hour or three-quarters of an hour before they let me go, yes.* (0014–00021_IV-SC_Part C_CRC) *Not at all [no concerns]. We did that all prior to the injection. Yes, we did [have enough time]. I did that back in the doctor’s surgery prior to getting everything official with signing your bits of paper that was then I [was] able to talk with the doctor and he with me and learn what was going to happen.* (0015–00115_SC_Part D_RCC)
*Question 5: Did the length of time to administer the study medication impact the amount of time you had to interact or socialize with other individuals besides your nurse or doctor? a) Not at all b) A little bit c) Somewhat d) Quite a bit e) Very much f) Not applicable	No impact	N = 23 of 23 (100)	*That was not really an issue. I mean, everyone was busy with their own treatment. I just was concentrating on my own and hoping there wouldn’t be any reaction to the injection. [SC] not really [affect the time with others]. But no, it didn’t really affect anything at all.* (0014–00113_SC_Part D_CRC)
*Question 6: How bothered are you about the amount of time it took for your study medication to be injected/infused? a) Not at all b) A little bit c) Somewhat d) Quite a bit e) Very much	No bother	N = 30 of 33 (91)	*Don’t really have a problem with it [the time it took to get the treatment], no. It would be nice if it was shorter, but if that’s what it takes, that’s what it takes [laughter].* (0018–00028_IV-SC_Part C_NSCLC) *No, no, no, I didn’t feel bothered at all… sometimes if you were sitting down for a while, you just feel back pain and you have to work a little bit. but it’s not important. When you have been sitting down for a while, it’s uncomfortable but nothing extreme.* (0016–00103_SC_Part D_RCC)
	Some bother	N = 3 of 33 (9.0)	*The injection is fast, but it takes some time waiting for the injection from the pharmacy.* (0025–00107_SC_Part D_NSCLC)
**Question 7:** Overall, how satisfied or dissatisfied are you with how your study medication was administered? a) Very satisfied b) Somewhat satisfied c) Neither satisfied or dissatisfied d) Somewhat dissatisfied e) Very dissatisfied	**Total sample (N = 43):** Rating of 9.4	N = 43 (100)	*I’m really satisfied I would say. I’ll even give it a 9. It just seems so easy. There’s no problem, there’s no having to sit there for 2 or 3 hours with a needle in your arm. Everything just seems to flow with it. It’s a great way of having… it put into your body.* (0014–00021_IV-SC_Part C_CRC) *10… because it was painless and really non-invasive. Of course, it was an injection into my abdomen but compared to the chemo, it was just like having a little butterfly move across your tummy.* (0014–00113_SC_part D_CRC)
*Question 8: If given the choice, which route of administration for your study medication would you prefer? [Part C participants only, N = 11] a) Intravenous infusion b) Subcutaneous injection c) No preference	SC administration	N = 10 (90.9)	*Oh, I’d prefer the sub. The subcutaneous treatment is a lot of easier. [because] you’re not having to have the hassle of sitting there for an hour while you have the treatment.* (0014–00015_IV-SC_Part C_RCC) *Yeah, oh, well, subcutaneous then, yes, clearly. first of all, it’s much quicker to administer. [The IV] really does take a lot of time and then you have to just wait, at least with the syringe, once it’s prepared, they administer it immediately and I do think that is a much more pleasant experience.* (0039–00084_IV-SC_Part C_HCC)
	IV administration	N = 1 (9.1)	*Well, IV, in fact, the detail is when they do the subcutaneous I have to stay 30 min, no, 1 hour afterward to see if there are no problems. When it’s IV, it’s 30 min of injection and then I can go home directly. I prefer the 30 min and go direct rather than 1 min, 5 min, and staying 1 hour.* (0021–00075_IV-SC_Part C_CRC)

Not every question was asked of every sub-study interview participant. In these instances, the rows for a given question may add up to less than n = 43.

Abbreviations: IV, intravenous; SC, subcutaneous; Tx, treatment.

#### 3.2.1 Pain severity (PEPQ question 1)

Severity of pain (relevance of PEPQ Q1 concept) was a common response when participants were asked to describe symptoms related to the IV infusion or SC injection. Many participants (*n* = 26/43, 60.5%) did not experience pain and described the IV infusion or SC injection as “pain-less,” “not painful,” or “no pain.” More than one-third of participants (*n* = 17/43, 39.5%), however, reported a range of pain severity experiences related to the IV infusion or SC injection, notably “pricking,” “stinging” (*n* = 5/43, 11.6%, each), “burning” or “tenderness” (*n* = 3/43, 7.0%, each), “ache or dull sensation” or “pain” (*n* = 2/43, 4.7%), and “discomfort” or “pinching” (n = 2/43, 2.3%, each) (Table 3).

**TABLE 3:** Symptom experience immediatiely after infusion/injection.

Symptom/sign description	Total, N (%)*
**Injection-related symptom/sign**	28 (65.1)
Redness	12 (27.9)
Itching	6 (14.0)
Tingling	5 (11.6)
Pricking pain (of needle)	5 (11.6)
Stinging	5 (11.6)
Lump	3 (7.0)
Burning	3 (7.0)
Tenderness	3 (7.0)
Warm or hot sensation	3 (7.0)
Ache or dull sensation	2 (4.7)
Pain	2 (4.7)
Cold sensation	2 (4.7)
Discomfort	1 (2.3)
Pinching pain	1 (2.3)
Discoloration	1 (2.3)
Irritation	1 (2.3)
Swelling	1 (2.3)
Thickening	1 (2.3)
Did not report any sensations	15 (34.9)
**General pain and discomfort**	**2 (4.7)**
Joint pain	1 (2.3)
Numbness	1 (2.3)
**Digestive symptoms**	**3 (7.0)**
Change in appetite	1 (2.3)
Change in taste	1 (2.3)
Vomiting	1 (2.3)

*Reported during and/or after injection/infusion.

#### 3.2.2 Symptoms experienced (PEPQ question 2)

Other symptoms reported by participants (relevance of PEPQ Q2 concept) related to the IV infusion or SC injection included skin-related symptoms or signs. Two-thirds of participants (*n* = 28/43, 65.1%) described skin-related symptoms or signs during or immediately after IV infusion or SC injection. A third of participants (*n* = 15/43, 34.9%) did not report any other non-pain symptoms or signs. The most common skin-related symptoms described were “redness” (*n* = 12/43, 27.9%), “itching” (*n* = 6/43, 14.0%), and “tingling” (n = 5/43, 11.6%). A few individuals reported general body pain, body discomfort, or digestive symptoms (*n* = 3/43, 2.3%; one each reported) but did not attribute them specifically to the study medication or the route of administration. Descriptions of symptom severity associated with SC injections were not substantially different among participants from parts C, D, or E.

#### 3.2.3 Time for injection/infusion (PEPQ question 3)

The length of time for the administration of treatment (relevance of PEPQ Q3 concept) is an important dimension contributing to how participants may feel about the route of administration. Participants reported the length of time for the SC injection as “less than 5 min,” “short amount of time,” or in terms of whether it was “faster” or “slower” than what they expected. Almost all sub-study participants (*n* = 42/43, 97.7%) responded that it took less time than what they expected (*n* = 19/42, 45.2%), about the same as expected (*n* = 17/42, 40.5%), or their SC treatment took more time than expected (*n* = 6/42, 14.3%).

#### 3.2.4 Amount of bother about time for injection/infusion (PEPQ question 6)

Similarly, when describing the level of time burden (relevance of PEPQ Q6 concept) for the IV infusion or SC injection, three-fourths of participants (*n* = 33/43, 76.7%) described not being bothered (*n* = 30/33, 91%) about the time it took. Those that experienced some bother (3/33, 9.09%) mentioned that they were bothered by the time it takes to prepare the medicine and waiting for it to be administered.

#### 3.2.5 Impact on communication with a nurse or doctor (PEPQ question 4)

The experience of satisfaction with the route of administration (relevance of PEPQ Q4 concept) can be influenced by the level of communication between the patient and the nurse or doctor. Generally, participants described having “plenty of time to ask questions,” “enough time to talk,” or felt “taken care of” by their care team. All of the interview participants (*n* = 43/43, 100%) believed that the IV infusion or SC injection had no impact on the amount of time available to speak with a nurse or doctor about questions or concerns about their illness or the treatment.

#### 3.2.6 Time to socialize (PEPQ question 5)

Another facet for evaluating experience with the route of medication administration is the amount of time patients have to socialize with fellow patients or clinic staff (relevance of PEPQ Q5 concept). Twenty-three participants (*n* = 23 of 43, 53.5%) reflected on the time available during the IV infusion or SC injection visit to socialize. Participants who reflected on this question (*n* = 23/23, 100%) described “talk[ing] to everyone,” having “heaps of time,” “plenty of time to talk” to people, or finding that time “has not been a problem.”

#### 3.2.7 Overall satisfaction with administration of medication (PEPQ question 7)

When thinking about overall satisfaction with the administration of the medication, participants considered their overall experience with the study treatment (relevance of PEPQ Q7 concept). Part C participants reflected on both IV and SC experiences, whereas parts D and E participants reflected on SC experience. Participants were asked to consider the route of administration of the treatment, the amount of time the treatment took, whether the medication worked for them, the degree of invasiveness of the administration (e.g., finding a vein for IV administration), and the amount of pain of the administration. Overall satisfaction with the route of medication administration was very high. The majority (*n* = 41/43, 95.3%) described the route of medication administration as being “very good” and feeling “very satisfied.” Two participants did not directly answer the question.

Table 4 shows a summary of the satisfaction ratings and responses to the final preference question in the interview guide. Ratings of treatment satisfaction were reported by participants using an 11-point (0–10) numerical rating scale (NRS). The results for the overall satisfaction rating of the route of administration of treatment were rated very high across parts C, D, and E participants. The total group mean for “satisfaction with SC treatment experience” was 9.4 on the NRS.

**TABLE 4:** Satisfaction with treatment experience and preference for the route of medication administration.

Satisfaction with treatment experience (NRS scale 0–10)	Parts C, D, and E participants (N = 43)
Mean (SD)	9.4 (0.9)
Median (Range)	10 [7 to 10]
**Preference for the route of medication administration, N (%)**	**Part C participants (N = 11)**
Preferred SC administration*	10 (90.9)
Preferred IV administration	1 (9.1)
No preference	0 (0.0)

*Note: The parts D and E participants did not have IV administration, so they did not provide any data for the preference question.

Abbreviations: IV, intravenous; NRS, numerical rating scale; SC, subcutaneous; SD, standard deviation.

#### 3.2.8 Preference for the route of medication administration (PEPQ question 8)

Preference for the route of medication administration (relevance of PEPQ Q8 concept) was asked specifically of part C participants (*n* = 11/43, 25.6%) as they had recent experience within the trial of both routes of study medication administration. All but one of the part C participants (*n* = 10/11, 90.9%) preferred the SC administration over IV administration. Those that preferred the SC route of administration described it as “comfortable,” “less invasive,” “less painful,” and “less aggressive.” The one participant who preferred IV indicated that it was due to the length of monitoring time after the SC administration in the clinic.

### 3.3 Revisions to PEPQ v1.0

After the piloting of the PEPQ during the CA209-8KX trial, the PRO development team decided to remove question 2 “feelings or sensations during the injection or infusion” and revise the instrument from interviewer-administered to self-report PRO instrument (see Supplemental Table S1). The rationale to remove question 2 was multi-fold. One strong rationale was due to the perceived burden of administering this questionnaire from clinical site staff. A second compelling rationale was the potential for missing data, given dependence on the interviewer to accurately record responses. A third rationale for removing question 2 was that question 1 (IV/injection pain) captures key symptom experience related to routes of administration, whereas other symptoms may be more related to the treatment itself.

The sub-study interview feedback supported this decision to remove question 2 as the key symptoms reported by participants related to IV infusion or SC injection were primarily specific to pain which is captured in PEPQ question 1. Removing question 2 would simplify for patient completion rather than interviewer-administration and allow for easier scoring and interpretation of the PEPQ. The revised PEPQ v2.0 includes seven questions, and the instructions have been revised for self-report.

## 4 Discussion

For this study, embedded interviews offered an opportunity to assess the relevance of concepts encompassed in the PEPQ from the target population of CA209-8KX trial participants. Satisfaction with treatment was high for the interviewed participants, and the participant feedback demonstrated overall positive experience with the route of medication administration The results mapping interview feedback on the experience of IV and SC routes of study medication administration to the concepts in the PEPQ demonstrate that pain symptoms and impacts to time are relevant and valid concepts for assessing satisfaction for routes of medication administration. Feedback from participants did not identify any missing concepts not captured in the PEPQ. The mixed-methods approach in this analysis allows for the evaluation of convergence or cross-validation between methods. The interview results support the concepts covered by the PEPQ.

A 2021 review of published studies describing preferences for IV or SC treatment in patients with chronic disorders assessed concepts such as time requirement, convenience, side effects, and fear of injections (Overton et al., 2021). Research on cancer patient satisfaction has focused on satisfaction with care which focuses on the context of the treatment, rather than on homing in on the route of medication administration (Brédart et al., 2010). SC administration can reduce time spent waiting for and receiving treatment as it shortens injection times, removes the need for IV infusion ports, decreases the time spent, reduces provider and facility time to deliver cancer care, and offers cost-saving advantage compared to IV.

The PEPQ provides a 7-item tool for use in oncology clinical trials that can provide valuable information on patient satisfaction for the route of medication administration. Future studies may focus on further validation of the PEPQ, assessing the incidence of adverse events between routes of administration, and evaluating the humanistic and economic impact of SC *versus* IV administration.

## 5 Limitations

When interpreting the current content validity study, some limitations should be considered. The PEPQ v1.0 was developed from a targeted literature review of satisfaction measures and consultation with clinical experts as an interviewer-administered measure to assure the completion of the symptom checklist and open-ended responses. Ideally, *de novo* PRO development should begin with patient input and follow the Food and Drug Administration (FDA) guidance (Food and Drug Administration, 2022a; Food and Drug Administration, 2022b).

The PEPQ was not evaluated directly through cognitive interviews but examined by mapping question concepts from the PEPQ to the participant feedback and experiences on topics in the interview guide. No direct feedback or assessment of the PEPQ was obtained. However, the sub-study probed on PEPQ concepts, and support was found for all questions in the PEPQ from the range of topics that participants brought up as relevant to their experience of the treatment and route of administration. There was no evidence of missing concepts for assessing the experience during or directly after IV infusion or SC injection.

Additional limitations should be considered when reviewing these results. Only part C participants (*n* = 28) were administered the entire PEPQ v1.0 as a part of their study assessments. Part C participants were the only group that had direct experience receiving the treatment *via* both IV and SC routes of administration. Parts D and E participants received an SC injection during the trial; however, they may not have had exposure to IV in prior treatment before enrolling into this study. From the clinical trial findings (Lonardi et al., 2022), 21 part C participants (*n* = 21/28, 75.0%) responded on the PEPQ, of which 15 (*n* = 15/21, 71.4%) indicated a preference for the SC route of medication administration. In comparison, 10 (*n* = 10/11, 90.9%) of the part C participants in the qualitative interview sample indicated a preference for SC treatment. There are several potential explanations for this difference. One explanation for the difference preference for SC treatment between the part C trial and interview sub-sample might be self-selection bias with those who agreed to interview also being participants who preferred SC injection over IV infusion. A second possibility could be a result of the difference in what participants might divulge to clinical site staff in a study visit *versus* a lengthy conversation with a qualitative interviewer. Another potential explanation might be that the interview is a retrospective discussion of overall clinical trial benefits and not a single point of time during a clinic visit. A fourth possibility might be that the difference is the result of a response shift due to time lapse (days or weeks) between when the interview participants completed the PEPQ and the interview. Regardless of these limitations, the qualitative interview results and the PEPQ trial results indicate the highest predominance of preference being for the SC route of administration.

Sub-study interviewers were trained on the semi-structured interview guide and asked to cover every probe included in the guide as much as reasonably possible for each telephone interview in the allotted interview time. However, due to the conversational nature of qualitative research, some probes may have been missed due to the natural flow of the discussion and dynamics with the participant and sometimes due to time restraints.

Further limitations of this study resulted from the questionnaire mode of administration of PEPQ v1.0, which was an interviewer-administered instrument. The clinical sites were responsible for administering the instrument to each trial participant. However, due to clinical sites’ other commitments and the additional burden of administering the PEPQ, missing data were noted. It is recommended that in future studies, the self-complete version of the PEPQ, PEPQ v2.0, be used to minimize clinical site staff administration burden and reduce the chances of missing data.

## 6 Conclusion

This study summarizes the experience, satisfaction, and preference for the route of medication administration between IV and SC treatment from a subgroup of participants from the CA209-8KX clinical trial with advanced NSCLC, RCC, melanoma, HCC, and CRC. Good concept convergence was noted for concepts related to the experience of IV or SC administration and the PEPQ items. These findings support the relevance and initial validity for the use of the PEPQ in oncology clinical trials.

## Data Availability

The raw data supporting the conclusion of this article will be made available by the authors, without undue reservation. Materials and data related to this study may be available upon request.
