# Supplementary material for: Patient experience of medication administration and development of a Patient Experience and Preference Questionnaire (PEPQ) for patients with advanced or metastatic cancer
Source: Front Pharmacol. 2024 Mar 27;15:1310546. doi: 10.3389/fphar.2024.1310546 (PMC11004258; doi:10.3389/fphar.2024.1310546)
Supplement: Supplementary file 1 [file Table1.pdf]

Supplementary Material

Patient Experience and Preference Questionnaire (PEPQ): Relevance of Content to Patients with Advanced or Metastatic Cancer

Anne M. Skalicky, MPH; Bryan Bennett, PhD; Judith Raimbourg, MD, PhD; Sara Lonardi, MD; Julia R. Correll, MPH; Iwona Lugowska, MD, PhD; Matthew Dixon, PharmD, PhD; Nashmel Sargalo, PhD; Mona L. Martin, RN, MPA

Supplemental Table 1. PEPQ Revisions

PEPQ v1.0 Question	Type of change	PEPQ v2.0 Question
Instructions for PEPQ:<br>• Please use the following directions for administering the Patient Experience and Preference Questionnaire to the study participant.<br>• Instructions for the individual administering the questionnaire are in the shaded boxes. Text in the shaded boxes should not be read aloud to the study participant.<br>• When reading the questions to the study participant, please make sure to speak clearly and slowly. Do not emphasize any words, as that could potentially influence the subject's response.<br>• Please make sure to read all questions exactly as written. If the participant has difficulty understanding the question, you may repeat the question, but please do not try to re-phrase or paraphrase the questions.<br>Record the responses directly from the participant directly on this form in the space provided. When recording responses, please make sure to capture their exact response.	Revised for self-report.	*Instructions for PEPQ:*<br>Record your responses directly on this form in the space provided.

PEPQ v1.0 Question	Type of change	PEPQ v2.0 Question
Question 1: Using a scale from 0 to 10, where 0 represents "No pain or discomfort at all" and 10 represents "Pain or discomfort that is as bad as you can imagine", please rate the overall amount of pain or discomfort that you experienced during the injection or infusion of the study medication today.	No change	Question 1: Using a scale from 0 to 10, where 0 represents "No pain or discomfort at all" and 10 represents "Pain or discomfort that is as bad as you can imagine", please rate the overall amount of pain or discomfort that you experienced during the injection or infusion of the study medication today.
Record the study participant's response below (check one box): 0 to 10	Revised for self-report.	Record your response below (check one box): 0 to 10
Instructions for Question 2:<br>Please do not read any of the words below.<br>• If a study participant mentions any of the words that are listed, please place a checkmark next to that word.<br>• If the study participant mentions a word that is similar, but not exactly the same, then do not place a checkmark next to the word, and instead write the alternative version of the term that the participant used next to the similar term.<br>• Please use the space on the right side to list any other terms that the participant used to describe his or her experience.<br>Check any terms that are mentioned (write alternative wording next to term)	Removed	Removed
Question 2: Please tell me any words that you would use to describe any feelings or sensations that you may have experienced at any point during the injection or infusion of your study medication today.	Removed	Removed
Response scale for question 2:<br>• Pain<br>• Warm<br>• Cold<br>• Burning<br>• Stinging	Removed	Removed

PEPQ v1.0 Question	Type of change	PEPQ v2.0 Question
• Itching<br>• Tingling<br>• Swelling<br>• Lump<br>• Tenderness<br>• Did not report any sensation<br>• Other (write other terms mentioned)		
Question 3: Which statement best describes the amount of time it took for your study medication to be injected/infused	No change	Question 2: Which statement best describes the amount of time it took for your study medication to be injected/infused?
Response scale for question 3:<br>a) Injection/infusion of the study medication took less time than I expected<br>b) Injection/infusion of the study medication took an acceptable amount of time<br>c) Injection/infusion of the study medication took longer than I expected	No change	a) Injection/infusion of the study medication took less time than I expected<br>b) Injection/infusion of the study medication took an acceptable amount of time<br>c) Injection/infusion of the study medication took longer than I expected
Question 4: Did the length of time to administer the study medication impact the amount of time you had to speak to your nurse or doctor about your illness or other concerns?	No change	Question 3: Did the length of time to administer the study medication impact the amount of time you had to speak to your nurse or doctor about your illness or other concerns?
Response scale for question 4:<br>a) Not at all<br>b) A little bit<br>c) Somewhat<br>d) Quite a bit<br>e) Very much<br>f) Not applicable	No change	a) Not at all<br>b) A little bit<br>c) Somewhat<br>d) Quite a bit<br>e) Very much<br>f) Not applicable
Question 5: Did the length of time to administer the study medication impact the amount of time you had to interact or socialize with other individuals besides your nurse or doctor?	No change	Question 4: Did the length of time to administer the study medication impact the amount of time you had to interact or socialize with other individuals besides your nurse or doctor?

PEPQ v1.0 Question	Type of change	PEPQ v2.0 Question
Response scale for question 5:<br>a) Not at all<br>b) A little bit<br>c) Somewhat<br>d) Quite a bit<br>e) Very much<br>f) Not applicable		a) Not at all<br>b) A little bit<br>c) Somewhat<br>d) Quite a bit<br>e) Very much<br>f) Not applicable
Question 6: How bothered are you about the amount of time it took for your study medication to be injected/infused?	No change	Question 5: How bothered are you about the amount of time it took for your study medication to be injected/infused?
Response scale for question 6:<br>a) Not at all<br>b) A little bit<br>c) Somewhat<br>d) Quite a bit<br>e) Very much	No change	a) Not at all<br>b) A little bit<br>c) Somewhat<br>d) Quite a bit<br>e) Very much
Question 7: Overall, how satisfied or dissatisfied are you with how your study medication was administered?	No change	Question 6: Overall, how satisfied or dissatisfied are you with how your study medication was administered?
Response scale for question 7:<br>a) Very satisfied<br>b) Somewhat satisfied<br>c) Neither satisfied or dissatisfied<br>d) Somewhat dissatisfied<br>e) Very dissatisfied	No change	a) Very satisfied<br>b) Somewhat satisfied<br>c) Neither satisfied nor dissatisfied<br>d) Somewhat dissatisfied<br>e) Very dissatisfied
Question 8: If given the choice, which route of administration for your study medication would you prefer?	No change	Question 7: If given the choice, which route of administration for your study medication would you prefer?
Response scale for question 8:<br>a) Intravenous infusion<br>b) Subcutaneous injection	No change	a) Intravenous infusion<br>b) Subcutaneous injection<br>c) No Preference

PEPQ v1.0 Question	Type of change	PEPQ v2.0 Question
c) No Preference		

Abbreviations: NA = not applicable; PEPQ = Patient Experience and Preference Questionnaire
